# Enhanced High-Resolution and Long-Range FMCW LiDAR with Directly Modulated Semiconductor Lasers

**DOI:** 10.3390/s25134131

**Published:** 2025-07-02

**Authors:** Luís C. P. Pinto, Maria C. R. Medeiros

**Affiliations:** 1 Instituto de Telecomunicações (IT), 3030-290 Coimbra, Portugal; carlos.pereira@co.it.pt; 2 Department of Electrical and Computer Engineering (DEEC), University of Coimbra, 3030-290 Coimbra, Portugal

**Keywords:** FMCW LiDAR, semiconductor laser, chirp linearization

## Abstract

Light detection and ranging (LiDAR) sensors are essential for applications where high-resolution distance and velocity measurements are required. In particular, frequency-modulated continuous wave (FMCW) LiDAR, compared with other LiDAR implementations, provides superior receiver sensitivity, enhanced range resolution, and the capability to measure velocity. Integrating LiDARs into electronic and photonic semiconductor chips can lower their cost, size, and power consumption, making them affordable for cost-sensitive applications. Additionally, simple designs are required, such as FMCW signal generation by the direct modulation of the current of a semiconductor laser. However, semiconductor lasers are inherently nonlinear, and the driving waveform needs to be optimized to generate linear FMCW signals. In this paper, we employ pre-distortion techniques to compensate for chirp nonlinearity, achieving frequency nonlinearities of 0.0029% for the down-ramp and the up-ramp at 55 kHz. Experimental results demonstrate a highly accurate LiDAR system with a resolution of under 5 cm, operating over a 210-m range through single-mode fiber, which corresponds to approximately 308 m in free space, towards meeting the requirements for long-range autonomous driving.

## 1. Introduction

Light detection and ranging (LiDAR) technology has gained prominence across various applications due to its ability to provide precise distance measurements and the detailed identification of surfaces and textures. In autonomous driving, LiDAR plays a critical role in object detection, environment mapping, and ensuring safe navigation [1,2]. In vegetation analysis, it is used to generate detailed land models that incorporate vegetation structures, supporting advancements in agriculture, ecological research, and urban greening initiatives [3]. Additionally, in transportation infrastructure, LiDAR contributes to road construction and maintenance by producing high-resolution maps that are essential for planning, monitoring, and optimizing roadway projects [4]. LiDAR is also gaining relevance in Industry 4.0, where it enables the precise monitoring of industrial environments, enhances automation processes, and supports real-time decision making in smart manufacturing systems [5].

LiDAR systems are generally based on two main technologies: time-of-flight (ToF) and frequency-modulated continuous wave (FMCW) [6]. While ToF LiDAR systems are widely used, they present limitations such as reduced sensitivity and compromised precision and stability in environments with optical interference. In contrast, FMCW LiDAR systems offer superior performance in long-range measurements by leveraging coherent detection. Additionally, unlike ToF systems, FMCW LiDAR enables direct velocity measurement, providing an advantage in dynamic sensing applications [6].

Key figures of merit for FMCW LiDAR systems include high-range resolution, a long detection range, rapid scanning rates, and minimal post-processing complexity [7]. Meeting these performance targets demands an optical source with carefully engineered properties: a broad frequency sweep bandwidth to achieve fine-range resolution, a narrow instantaneous linewidth to maintain coherence over extended distances, a fast and stable chirp rate to enable high-speed measurements, and highly linear frequency modulation without mode hopping to ensure accurate distance retrieval and streamline signal processing.

The broad fast linear frequency chirp is typically achieved by two main approaches. The first one uses a continuous wave (CW) narrow linewidth laser followed by an external optical modulator [8,9], which is modulated by a linear radio frequency (RF) FMCW signal with the same bandwidth as the required optical chirp bandwidth. Although this approach generates a high-quality FMCW optical signal, it relies on costly high-bandwidth RF and photonic components. A simplest alternative is to generate an optical chirp signal by direct modulation of a semiconductor laser [10].

The origins of chirp nonlinearity of a directly modulated semiconductor laser can be divided into two types: random and systematic [11]. Random nonlinearity arises from unpredictable external factors, such as temperature fluctuations or spontaneous emission within the laser. These factors introduce uncontrolled variations that can affect both the frequency and the phase of the laser. Systematic nonlinearity is the nonlinear behavior that becomes repetitive and after characterized, its behavior can be measured and compensated by using specific techniques. To address these nonlinearities, various strategies have been proposed. Strategies with the necessity of greater computing power like post processing strategies were employed, such as the resampling method [12,13]. Methods with more complex hardware such as optical phase-locked loop (OPLL) also have demonstrated good performance [14,15].

The iterative learning control (ILC) method proposed in [16] offers a simple yet effective approach to achieve a linear frequency sweep through pre-distortion techniques. This method is both flexible and robust, as it can be applied to any laser without requiring prior knowledge of its specific characteristics. The method has been successfully applied with a fast convergence time in [17] and applied in the Fourier domain [18]. In this paper, we follow closely [16,17]; however, we have introduced a reference slope that adapts at each interaction and cross-correlation of individual scan periods for improved time alignment, achieving improved sweep linearity. While the bandwidth of 3.28 GHz and theoretical resolution of 4.57 cm are lower compared to [16,17], our achieves a notably high repetition rate of 55 kHz, enabling faster measurement cycles. Furthermore, it demonstrates an extended measured distance capability of 210 m, surpassing previous works and highlighting enhanced robustness and operational range. The residual nonlinearity of 0.0029% and the low correlation error $1-r^{2}=8.1\times 10^{-8}$ confirm the superior linearization performance.

In this paper, the frequency chirp dynamics of directly modulated DFB lasers are examined in detail, highlighting the dual influence of fast carrier-induced effects and slower thermal processes. The carrier-induced chirp is linked to rapid changes in optical power, while thermal chirp arises from self-heating and exhibits a delayed response due to the thermal inertia of the active region. The relative dominance of each mechanism depends on the modulation frequency: thermal effects prevail at low frequencies (typically below 10 kHz), whereas carrier effects dominate at higher frequencies.

The rest of the paper is structured as follows: Section 2 explores the principles of FMCW LiDAR for measuring distances and speeds. Section 3 analyzes the nonlinearity of optical chirps in semiconductor lasers and their impact on range resolution. Section 4 details the experimental setup to evaluate this nonlinearity, complemented by experimental results. Section 5 presents a chirp linearization method using ILC pre-distortion and demonstrates the achieved linearization results. Section 6 evaluates the effectiveness of linearization with optical fiber distance measurements. Finally, Section 7 summarizes the main conclusions.

## 2. FMCW LiDAR Fundamentals

**Figure 1:**
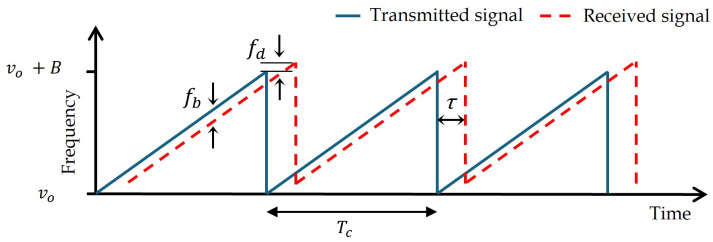
Frequencies of the reference beam (solid line) and the beam reflected by a distant target (dashed line).

The principle of a coherent FMCW LiDAR is based on a linear chirped optical signal $x_{i}\left(t\right)$, whose instantaneous frequency sweeps from an optical carrier frequency $\upsilon_{0}$ over a bandwidth *B* during a sweep time $T_{c}$, as illustrated in Figure 1 [19]. Despite the availability of more complex linearly chirped waveforms, such as the triangular waveform, the sawtooth is most commonly used to introduce the fundamental concepts of FMCW operation due to its simplicity.

(1) $$x_{i}\left(t\right)=\cos\left(2\pi \;\upsilon_{0}\;+\frac{B\;t^{2}}{T_{c}}\right)$$

**Figure 2:**
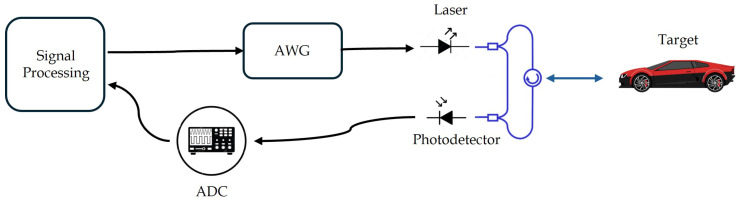
Basic configuration of an FMCW laser LiDAR.

As illustrated in Figure 2, this signal emitted by the laser is split into two paths: a reference path which acts a local oscillator and a probe path directed towards the target at a distance (range) *R*, moving at a radial velocity of *V*. Ignoring the channel response and assuming ideal target reflectivity, the reflected signal $x_{r}\left(t\right)$, is considered to be a delayed version of the transmitted signal, with a time delay $\tau =\frac{2(R\;+\;Vt)}{c}$, where *c* is the speed of light. At the photodetector, the transmitted signal and the delayed reflected signal are optically mixed, producing an electrical signal $x_{b}\left(t\right)=x_{i}\left(t\right)\;x_{r}\left(t\right)$. The mixing process results in two frequency components. The lower frequency term, the difference of the frequencies of both signals, corresponds to the beat frequency $f_{b}$, which contains information about the target’s range and velocity. The second term, the sum frequency, lies well beyond the photodetector bandwidth and is filtered out. At each sweep the target range can be calculated as

(2) $$R=\frac{f_{b}\;c\;T_{c}}{2\;B}$$

while the target velocity is calculated by the Doppler frequency shift $f_{d}$:

(3) $$V=\frac{f_{d}\;c}{2\;\upsilon_{0}}$$

**Figure 3:**
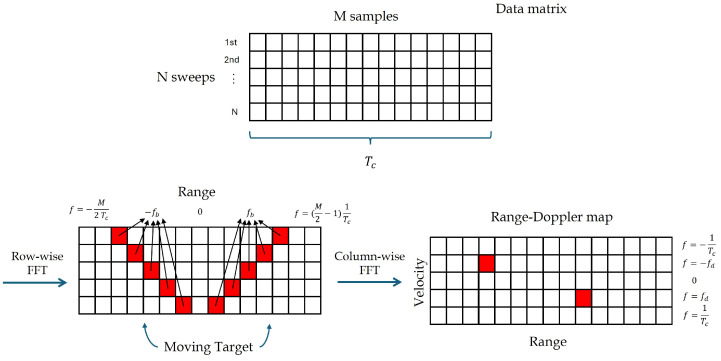
FMCW signal 2D FFT processing.

$f_{b}$ and $f_{d}$ are calculated from the digitalized $x_{b}\left(t\right)$, which is sampled by an analog-to-digital converter (ADC) operating at a sampling frequency of $f_{s}$, and then processed by the signal processing unit. For the digital signal processor (DSP), $x_{b}\left(t\right)$ is represented by the N×M matrix, illustrated in Figure 3, where *N* is the number of sweeps and *M* is the number of samples in each sweep. The number of samples in each sweep is $M=T_{c}\;f_{s}$. A two-dimensional (2D) fast Fourier transform (FFT) is used to process the signal.

The first FFT is performed along each row of the matrix, i.e., across each individual sweep. This yields *M* spectral bins, which, after applying an FFT shift, range from $\frac{-f_{s}}{2}$ to $\frac{f_{s}}{2}-\frac{f_{s}}{M}$, with a frequency resolution (bin spacing) of $\Delta f=\frac{f_{s}}{M}$. The beat frequency corresponds to the frequency where maximum value of the FFT is obtained. The range resolution depends solely on the chirp bandwidth and is given by

(4) $$\Delta R=\frac{\Delta f_{b}\;c\;T_{c}}{2\;B}\;=\frac{c}{2\;B}$$

While the maximum range is

(5) $$R_{max}=\frac{c\;M}{4\;B}$$

To calculate velocity, the Doppler shift is calculated by a second FFT, having as input the output of the first FFT over one chirp period, over the *N* consecutive chirp periods. Each column is a *N* point time series with sampling time $T_{c}$. $f_{D}$, corresponds to the frequency where maximum value of this second FFT is obtained. Doppler shift is calculated with a resolution of $\Delta f_{D}=\frac{1}{N\;T_{c}}$, which results in a velocity resolution of $\Delta V=\frac{c}{2\;\upsilon_{0}\;N\;T_{c}}$, and maximum measurable velocity $V_{max}=\frac{c}{4\;\upsilon_{0}\;T_{c}}$.

We note that the signal processing approach must take into account the shape of the modulation waveform. In particular, triangular modulation affects velocity measurements, as it requires processing Doppler shifts from both the up and down chirps to accurately resolve both speed and direction. This is in contrast to sawtooth modulation, which has a unidirectional frequency sweep per cycle and simplifies Doppler analysis. Nevertheless, the core principle for range estimation remains the same regardless of the modulation waveform.

## 3. Optical Frequency Chirp of a Directly Modulated DFB

The optical frequency of a DFB laser is governed by the refractive index of its active region, which is influenced by two main mechanisms: carrier density, varying on a fast (sub-nanosecond) timescale, and temperature, varying more slowly (microsecond to millisecond scale). As the injection current changes, it modulates both the carrier density and consequently the optical power, P(t), leading to time-dependent frequency shifts. The frequency deviation due to carrier dynamics (adiabatic and dynamic chirp), $\Delta \upsilon_{c}\left(t\right)$, as function of P(t), is given by [20]

(6) $$\Delta \upsilon_{c}\left(t\right)=\frac{\alpha }{4\pi }\left[\frac{1}{P(t)}\frac{\delta P(t)}{\delta t}+k\;P\left(t\right)\right]$$

where α and *k* are the linewidth enhancement factor and the adiabatic chirp scaling factor, respectively.

The optical output power P(t) does not respond instantaneously to variations in the injection current. Instead, it is limited by the carrier lifetime, which introduces a finite response time in the modulation of output power. Therefore, assuming a linear and immediate relationship between the drive current and optical power is only valid when the modulation bandwidth is well below the inverse of the carrier lifetime. Thermal effects cause an additional frequency shift due to self-heating in the active region [21].

This thermal chirp can be modeled as

(7) $$\Delta \upsilon_{th}\left(t\right)=-K_{th}\left(\int_{t}^{0}\frac{P(\tau )}{\tau_{th}}e^{-\frac{\left(t-\tau \right)}{\tau_{th}}}\;d\tau -\bar{P}\right)$$

where $\bar{P}$ is the steady-state power used as a reference, $K_{th}$ is the thermal chirp coefficient, and $\tau_{th}$ is the thermal time constant. This model accounts for the delayed response of the laser’s temperature to changes in optical power, reflecting the thermal inertia of the system. These dependencies highlight that both the current modulation amplitude and the laser’s thermal conditions play a critical role in determining the achievable chirp bandwidth; however, it is important to note that they act on distinct time scales.

Even when the case temperature is stabilized by a thermoelectric controller, fast local heating in the active region can occur faster than the controller can compensate. The relative contribution of thermal and carrier-induced chirp in a DFB laser depends on the modulation frequency with respect to the laser’s thermal time constant. The active region typically exhibits thermal time constants on the order of 10–100 μs, corresponding to thermal cut-off frequencies around 1–10 kHz. When the modulation frequency is below this range (e.g., <10 kHz), the laser has sufficient time to accumulate heat during each current cycle, leading to significant changes in refractive index and making the thermal chirp the dominant mechanism. Conversely, at higher modulation frequencies (e.g., >10 kHz), thermal effects cannot follow the rapid current variations, and frequency modulation is primarily governed by fast carrier-induced changes in the refractive index. As a result, the total chirp amplitude is larger at low frequencies due to thermal accumulation, and smaller at high frequencies where the response is limited to fast carrier dynamics. This introduces a trade-off: broader chirp bandwidths enable higher spatial resolution, while shorter chirp durations (corresponding to higher modulation frequencies) are required for accurate velocity measurements.

When a linear current ramp of duration $T_{c}$ is applied to the laser, a simplified yet effective phenomenological modeling approach is to represent the instantaneous optical frequency υ(t) as a combination of a linear and a nonlinear component, forming a black-box model [16]:

(8) $$\upsilon \left(t\right)=\upsilon_{0}+\frac{B}{T_{c}}t+\upsilon_{nl}\left(t\right)+\delta \upsilon_{sp}\left(t\right)$$

where $\upsilon_{0}$ is the optical carrier, $\frac{B}{T_{c}}t$ represents the ideal linear chirp, and $\upsilon_{nl}\left(t\right)$ captures the nonlinear frequency deviation.

The term $\delta \upsilon_{sp}\left(t\right)$ represents stochastic frequency noise induced by spontaneous emission, which introduces phase fluctuations and ultimately broadens the laser spectral linewidth, $\Delta v_{l}$. The plus sign corresponds to an ascending current ramp, while the minus sign applies to a descending ramp. In a coherent LiDAR, the frequency chirp nonlinearity manifests itself as a loss in resolution the nonlinearity-limited range resolution is given by

(9) $$\Delta R/R=2\pi (\upsilon_{nl,rms}/B)$$

where $\nu_{nl,rms}$ represents the RMS value of $\nu_{nl}\left(t\right)$ in the chirp period $T_{c}$.

An alternative to quantify the residual nonlinearity of the swept frequency laser is by the linear regression coefficient $1-r^{2}$, [16].

(10) $$\Delta R/R=2\pi \sqrt{(1-r^{2})/12}$$

In coherent LIDAR systems, the spectral linewidth of the laser also limits the maximum propagation distance due to the finite coherence length, given by $L_{coh}\;=\frac{c}{\pi \;\Delta \upsilon_{l}}$. Beyond this distance, the accumulated phase noise degrades coherent detection. However, successful signal recovery beyond the coherence length has been demonstrated [22,23].

## 4. Experimental Chirp Nonlinearity Assessment

**Figure 4:**
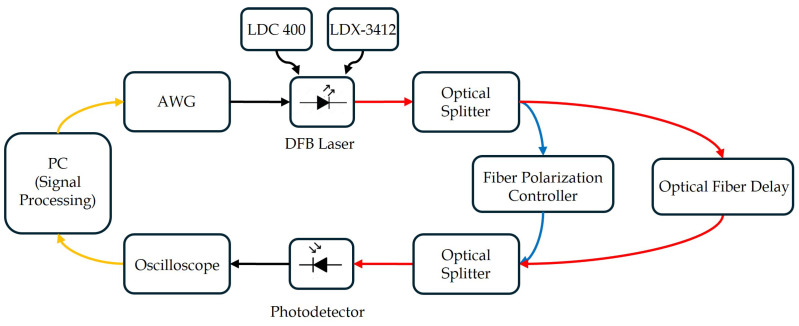
Schematic of the setup used for experimental tests and pre-distortion.

To observe the impact of the chirp nonlinearity, an experimental setup was designed and assembled, as illustrated in Figure 4.

The laser source used in the experiment was a DFB laser diode from the TOLD 387S-EAD Series (Toshiba, Tokyo, Japan), operating at a nominal wavelength of 1547 nm and with 2 MHz linewidth. To ensure thermal stability, the laser was temperature-controlled at 25.7 °C using a LDC 400 temperature controller (Thorlabs, Newton, NJ, USA). The device was biased at 54 mA via an ILX Lightwave LDX-3412 low-noise current source (Newport Corporation, Irvine, CA, USA). The emitted optical signal was split into two paths using a 50/50 fiber optic splitter: a reference path and a delayed path incorporating a 2-m single-mode fiber (SMF) delay line. A fiber-based polarization controller was inserted to maintain the polarization alignment between the two optical paths. The recombined signal was detected by a high-speed linear InGaAs optical receiver DSC-R402PIN (Discovery Semiconductors, Ewing Township, NJ, USA) with a 10 GHz bandwidth. The resulting electrical signals were recorded using a DPO3054 (Tektronix, Solon, OH, USA) digital oscilloscope, offering a 500 MHz analog bandwidth and a 2.5 GSa/s sampling rate.

A triangular waveform (55 kHz, 2 V_pp_) generated by a DG1022Z (RIGOL, Suzhou, China) arbitrary waveform generator operating at 55 MSa/s was used. Given the 50 Ω input impedance of the laser driver, this configuration produced a modulation current of 40 mA, resulting in sweep durations of 9.1 μs for up and down ramps.

**Figure 5:**
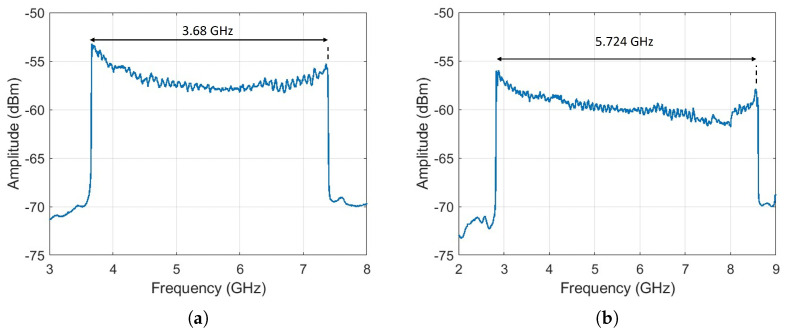
Spectra of the beat signal between modulated DFB laser and TLS laser with a similar wavelength. (**a**) 2$V_{\mathrm{pp}}$. (**b**) 3$V_{\mathrm{pp}}$.

The optical spectrum of the chirped signal was measured using an interferometric technique, where the chirped signal was mixed with a narrow-linewidth tunable laser source AP3350A (APEX Technologies, Marcoussis, France), 300 kHz linewidth). The measurement was performed with a spectral resolution of 100 kHz. As shown in Figure 5, when the applied electrical waveform has amplitudes of 2 V_pp_ and 3 V_pp_, the resulting chirp bandwidths are approximately 4 GHz and 6 GHz, respectively, confirming that increasing the modulation amplitude leads to a broader excursion of the optical power and, consequently, a wider frequency sweep, as predicted by Equation (6).

**Figure 6:**
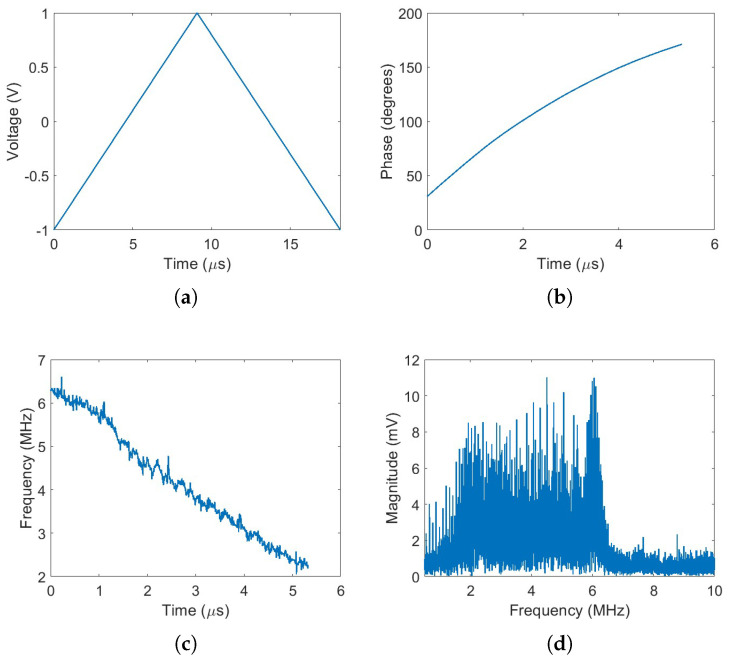
Laser modulation and signal analysis: (**a**) Applied laser drive voltage. (**b**) Phase of the up-ramp signal. (**c**) Instantaneous optical frequency of the up-ramp signal. (**d**) Spectrum of the beat signal.

The time–frequency curve (TFC) of the received beat signal was measured following the methodology proposed in [24]. To better observe the nonlinear behavior, only the up ramp is considered. Figure 6b shows the phase of the up ramp of the received beat signal after propagation over 2 m of optical fiber. As can be seen, the phase clearly deviates from linearity, while the calculated instantaneous frequency (Figure 6c) shows a 4.5 MHz variation. This frequency variations results in spectrum blur as can be seen in Figure 6d, the spectrum of the beat signal lacks a well-defined spectral peak, introducing significant uncertainty in determining the actual fiber delay between the two optical paths. These effects are direct consequences of the nonlinear frequency sweep of the laser.

## 5. Chirp Linearization for Enhanced LiDAR

**Figure 7:**
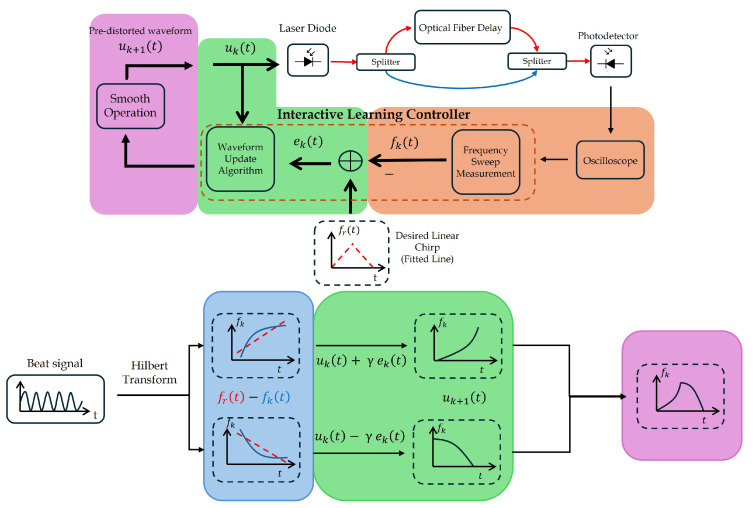
Schematic of the ILC pre-distortion for the laser chirp linearization.

We have developed a pre-distortion technique using an ILC which can be summarized in four steps, as illustrated in Figure 7, with each step color-coded for clarity.

The first step, marked in orange, is where the beat signal is captured by the oscilloscope. The instantaneous optical frequency of the laser chirp, $f_{k}\left(t\right)$, is reconstructed from the beat signal using a short fiber delay and phase analysis via the Hilbert transform as described in [24]. The TFC, $f_{k}\left(t\right)$, is the base curve responsible for the pre-distortion and quantification of nonlinearity. Although the optical source operates at a much higher carrier frequency, $\upsilon_{0}$, the analysis is performed in the RF domain, where $f_{k}\left(t\right)$ represents the instantaneous frequency derived from the beat signal. This representation captures the modulation behavior of the chirp and is sufficient to characterize and mitigate its linearity in FMCW LiDAR systems.

In the second step, the blue block, the error, $e_{k}\left(t\right)$, is calculated and, at the same time, the nonlinearity is quantified. The error is the difference between the desired linear chirp $f_{r}\left(t\right)$ and $f_{k}\left(t\right)$. This error captures the instantaneous deviation at each time point and is fundamental for the iterative correction process. Starting with $f_{r}\left(t\right)$, it is derived through a first-order polynomial fit to the measured $f_{k}\left(t\right)$. This means that a straight line is fitted to the TFC, which represents the ideal linear behavior that the $f_{k}\left(t\right)$ should follow. After having $f_{r}\left(t\right)$, the nonlinearity of $f_{k}\left(t\right)$ is quantified in terms of $1-r^{2}$.

In the third step, marked with green, the new input signal for the laser, $u_{k+1}\left(t\right)$, is generated iteratively using the linear ILC algorithm given by [25]

(11) $$u_{k+1}\left(t\right)=u_{k}\left(t\right)+\gamma \;e_{k}\left(t\right)$$

where γ is the learning gain. With this linear ILC algorithm, it was proven that $f_{k}\left(t\right)$ converges to $f_{r}\left(t\right)$ with a sufficiently small γ. As this is a fixed parameter, its optimal value must be carefully chosen.

In the last step, in the pink block, the update up and down ramps are subjected to smooth data to smooth the noise of the system. This is achieved using the *smoothdata* function of MATLAB with the *movmean* method, which applies a moving average filter to suppress high-frequency fluctuations and measurement artifacts. During the iteration process, both ramps are stored and used as input for the next iteration. The process repeats until the chirp linearity reaches a pre-established threshold, that we set to $10^{-9}$, or stops improving. In the end, both ramps are combined to form the final waveform.

Some modifications were introduced to the traditional pre-distortion methodology to improve flexibility and accuracy. Instead of using a fixed reference line, as described in [16,17], a linear least-squares fitting method with a first-order polynomial model was employed. This allows the reference slope to adapt across iterations, optimizing the process, particularly when focusing on a specific region of interest (ROI).

Furthermore, before distortion analysis, the acquired signal and consequently the ramps used for pre-distortion undergo pre-processing using a customized alignment algorithm. By using cross-correlation techniques, individual scan periods can be time aligned, filtering out poorly synchronized segments. The result of this is an optimized time-frequency average with significantly reduced timing jitter and measurement noise.

This dual approach, which combines adaptive reference optimization with signal conditioning, allows for the independent pre-distortion of up and down ramps, ultimately producing a highly linear frequency sweep with improved stability.

**Figure 8:**
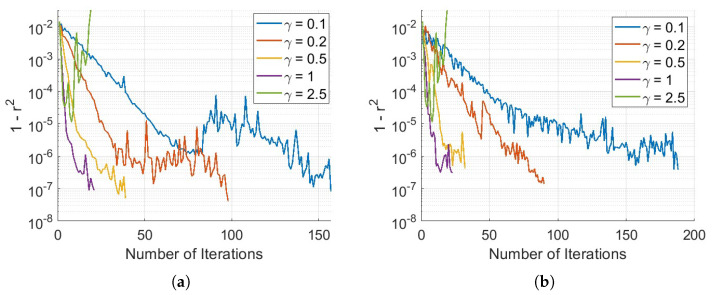
Residual nonlinearity, $1-r^{2}$, versus the number of iterations: (**a**) Up-ramp. (**b**) Down-ramp.

Figure 8 illustrate how the γ parameter influences the convergence behavior of $1-r^{2}$. A larger γ value accelerates convergence; however, a smaller γ is required to guarantee the stability and eventual convergence of the ILC process.

For the down ramp, it is evident that the γ value that leads to the best nonlinearity is γ=0.2. Except for γ=2.5, all other values achieve a $1-r^{2}$ in the range of $10^{-7}$. In the up-ramp, the values are very close, in the range of $10^{-8}$, except for γ=2.5, which causes the ILC algorithm to fail. With γ=0.5, it was possible to achieve low nonlinearity faster and with more consistent convergence.

As expected, a smaller γ value causes the algorithm to require more iterations to achieve a good nonlinearity value; however, it ensures that the process is more stable and less prone to oscillations. Better results were obtained in the up-ramp compared to the down-ramp, despite multiple attempts. This suggests that there may have been an error in data acquisition or instability during the down-ramp, as the same algorithm was applied to both.

**Figure 9:**
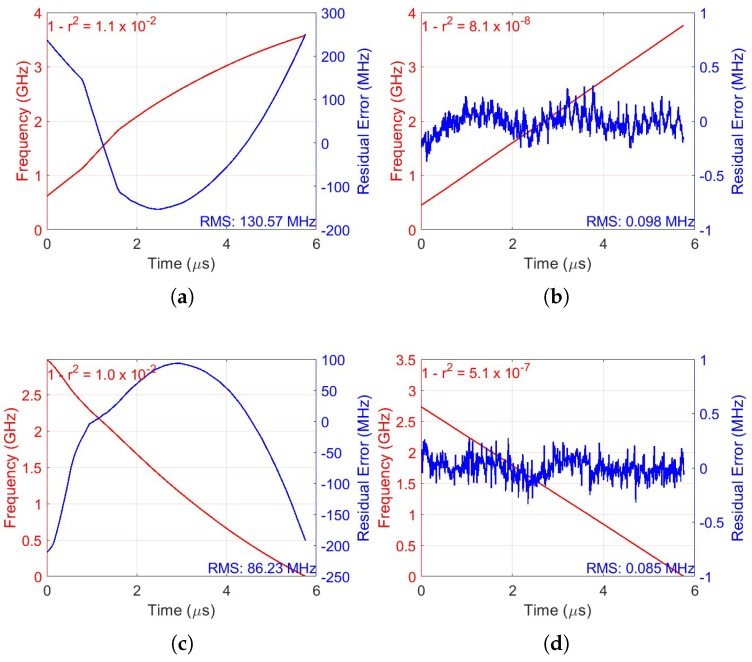
The up-ramp and down-ramp laser frequency sweep and residual error in the ROI, respectively. (**a**) 1st Iteration; (**b**) 95th iteration; (**c**) 1st iteration; and (**d**) 90th iteration.

Figure 9 illustrates the frequency sweeps and residual errors for the up-ramp and down-ramp at the 1st, 95th, and 90th iterations, respectively. The residual error was calculated from the difference between the real phase and the estimated linear phase, resulting in a phase deviation. This phase deviation was then converted into frequency deviation and is represented in blue in Figure 9. Two approaches were used for the frequency calculation. In the figures of the 1st iteration, Figure 9a,c, where the signal exhibits significant phase deviations resulting in a nonlinear frequency variation, the nonlinear swept frequency calculation was used to calculate the frequency [24]. With this approach, it is possible to capture the most noticeable distortions of the signal. For the 95th and 90th iteration figures, Figure 9b,d, since the signal is already pre-distorted and the phase variations are much smaller and more linear, the instantaneous frequency was estimated using the derivative of the phase extracted via the Hilbert transform. This approach is more suitable for signals where phase deviations are small, providing a more accurate frequency estimate.

With these two approaches, on the up-ramp, the nonlinearity was reduced from 130.57 MHz to 0.098 MHz, achieving a reduction factor of 1300, leaving a residual nonlinearity of only 0.0029%. Similarly, on the down-ramp, the nonlinearity decreased from 86.23 MHz to 0.085 MHz, corresponding to a reduction factor of 1000 and a residual nonlinearity of 0.0027%.

**Figure 10:**
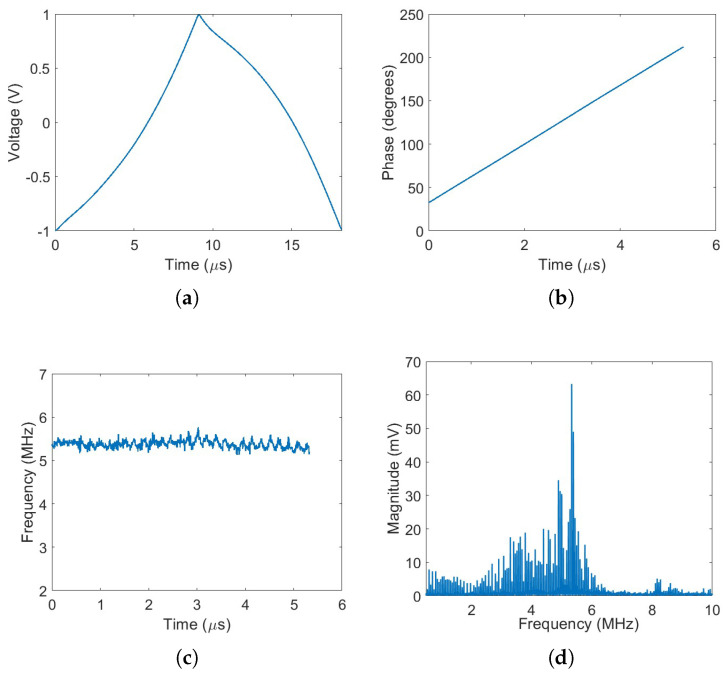
Pre-distorted signal generation and analysis: (**a**) Pre-distorted waveform produced by signal generator. (**b**) Phase of the up-ramp signal with pre-distortion. (**c**) Instantaneous optical frequency of the up-ramp signal with pre-distortion. (**d**) Spectrum of the beat signal.

Figure 10a presents the pre-distorted voltage waveform applied to the modulator. The phase of the resulting received beat signal is shown in Figure 10b, while the corresponding instantaneous frequency is depicted in Figure 10c. With the application of pre-distortion, the spectrum of the beat signal exhibits a clearly distinguishable peak, corresponding to the target distance, as shown in Figure 10d.

It is important to note that the ILC-based chirp correction is not performed in real time. Instead, it is an offline pre-distortion process, where the waveform is iteratively optimized prior to LiDAR operation. During this calibration phase, the system measures the frequency response of the laser, calculates the deviation from a linear chirp, and updates the modulation waveform over successive iterations. Once the desired linearity is achieved, the resulting pre-distorted waveform can be stored and used directly during real-time operation, without requiring further correction or computational effort. This approach enables real-time linear chirp generation with minimal system complexity, while leveraging the robustness and generality of the ILC learning scheme.

## 6. FMCW Range Measurement over Fiber

The experimental evaluation of the FMCW LiDAR system was conducted using an SMF delay cable to provide a controlled and repeatable optical path difference, simulating a fixed target distance. This approach enables the precise characterization of key performance parameters, such as beat frequency accuracy, chirp linearity, range resolution, and coherence properties, without the variability associated with free-space propagation. By introducing a known time delay, the fiber-based setup offers a stable environment for validating the LiDAR’s ranging accuracy and signal processing algorithms. This configuration ensures reliable and reproducible conditions for assessing the system’s overall performance.

The DFB laser was biased at 54 mA, and a pre-distorted modulation waveform with a peak-to-peak voltage of 2 V and a repetition rate of 55 kHz was applied. This modulation generated a chirp excursion of 3.28 GHz within the ROI, corresponding to a theoretical distance resolution of 4.57 cm. This approach avoids abrupt current transitions while maintaining consistency with the unidirectional sweep assumed in the theoretical model.

**Figure 11:**
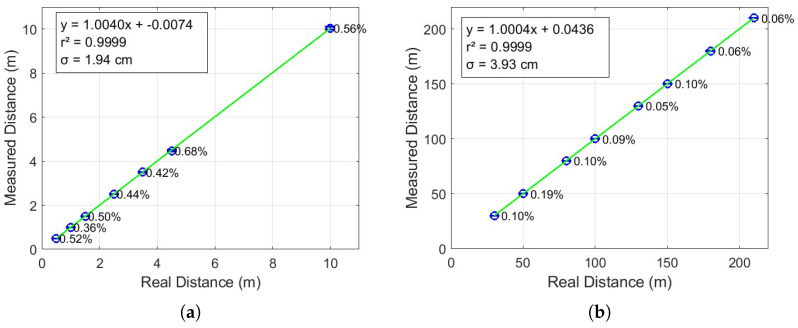
Fiber ranging test results: (**a**) From 50 cm to 10 m. (**b**) From 30 m to 210 m.

The measured fiber lengths versus the real fiber lengths are illustrated in Figure 11, with results presented in two separate plots. Figure 11a displays the short-range measurements, covering lengths from 50 cm to 10 m, with step sizes of 0.5, 1, and 5.5 m. Figure 11b shows the long-range measurements, ranging from 30 m to 210 m, in increments of 20 m. Each measurement was repeated ten times to improve accuracy and reduce the impact of random errors, enabling the calculation of reliable average values. In both figures, the blue circles represent the average measured distances relative to the actual distances. The blue error bars indicate the magnitude of the absolute errors, while the green line corresponds to the linear fit of the data. The equation of the fitted line, along with the coefficient of determination $r^{2}$, is displayed in the upper left corner of each plot to quantify the accuracy of the linear approximation.

The figure corresponding to short distances (0.5 to 10 m) in Figure 11a presents an excellent linear display ($r^{2}=0.9999$) between the real and measured distance [22,23]. The standard deviation is 1.94 cm, indicating that the system is stable and accurate at short distances. However, for longer fiber lengths, the percentage errors are progressively higher, varying between 0.36% and 0.68%. This is expected, given that small absolute variations have more percentage impact on short precision. Still, errors below 1% in these ranges are quite good and indicate that the system can measure with good fidelity even over short distances.

In cases of longer distances (30 to 210 m), represented in Figure 11b, the system maintains a very high linearity as well ($r^{2}=0.9999$), which reveals that the system’s operation is practically ideal. The observed standard deviation is 3.93 cm also below the theoretical resolution, which demonstrates robustness even at more demanding distances. The remarkably low percentage errors—less than 0.2%—show that the system is even more reliable in long ranges, where the relative impact of a small absolute error is smaller.

**Figure 12:**
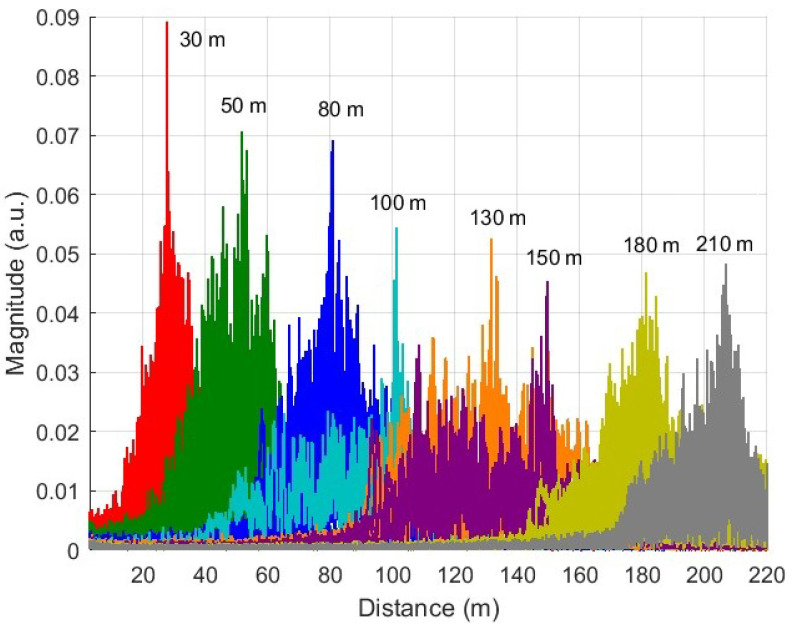
FFT analysis of beat signals for different distances.

Figure 12 shows the spectra of beat signals for different long-distance fiber lengths (30 to 210 m). Each peak in the graph represents the beat signal corresponding to a specific distance.

The results show distinguishable peaks for all distances, which indicates the system’s ability to detect different distances. Furthermore, we observe that the spectral broadening of the beat signal increases with fiber length, primarily due to laser decorrelation effects over long optical delays. However, since the laser that we are using has a 2 MHz, 210 m is well beyond the decorrelation distance.

The proposed method was compared with several pre-distortion techniques for FMCW LiDAR, as summarized in Table 1.

Starting with the method in [16], which shares the same ILC-based approach, our work outperforms in terms of residual nonlinearity (0.0029% vs. 0.0039%). Although not reported in [16], our work achieves a long measured distance (210 m), while operating at a much lower bandwidth (3.28 GHz vs. 36 GHz).

Comparing the adaptive iterative algorithm in [17], it manages to achieve a residual nonlinearity of 0.005%, and reports a distance of only 8 m. In our work, superior linearization is achieved and validated over distances 25 times greater, despite using lower modulation bandwidth and lower voltage levels.

The method in [26], which is based on linearization by interpolation, presents a greater nonlinearity (0.0236%) and is validated up to 100 m. In contrast, our proposed method offers a simpler implementation with competitive performance in range and chirp quality.

Furthermore, although the adaptive current injection method in [27] achieves slightly better linearity (0.0020%), it requires more complex hardware interactions and its effective range is not reported. In the Fourier domain method in [18], although it operates at a higher bandwidth (14.07 GHz), it offers a residual nonlinearity (0.0128%) more than four times worse than ours.

These comparisons highlight that our method provides an attractive balance between implementation simplicity, chirp linearization, and reachability, offering competitive or superior performance over more complex or bandwidth-intensive approaches.

## 7. Conclusions

In this paper, we proposed and experimentally validated a pre-distortion technique based on ILC to compensate for optical frequency chirp nonlinearity in FMCW LiDAR systems. The method iteratively corrects the waveform based on real system measurements, without requiring an accurate physical model, and is fully compatible with external modulation schemes used in photonic platforms. By applying the ILC-based pre-distortion to the measured waveform, we achieved a significant improvement in chirp linearity. The residual nonlinearity was effectively reduced across iterations, reaching relative levels below $10^{-8}$ after convergence, confirming the high effectiveness of the approach. In experimental distance measurements, the method enabled standard deviations of only 1.94 cm for target ranges between 0.5 m and 10 m, with percentage relative errors below 0.7%, and 3.93 cm for ranges between 30 m and 210 m, with percentage errors under 0.2%. These results highlight the strong correlation between chirp linearity and overall measurement precision. The proposed approach stands out for its generality, ease of implementation, and independence from a detailed physical system model, and is therefore applicable to a wide range of experimental configurations. This feature is particularly relevant for emerging LiDAR systems with integrated photonic platforms, in which small nonlinearities can strongly degrade system performance. While the ranging experiments in this work were conducted over single-mode optical fiber, the measured path lengths correspond to equivalent free-space distances of over 300 m. The use of fiber provided a highly stable and repeatable optical delay, enabling the precise evaluation of chirp linearity, coherence, and ranging resolution without the environmental variability introduced by free-space propagation. We acknowledge that real-world outdoor operation involves additional challenges, such as atmospheric turbulence, temperature gradients, beam divergence, mechanical alignment stability, and background light interference, which were beyond the scope of this initial study. Addressing these channel effects will require significant future work in system-level design, packaging, and environmental robustness. We are currently developing an outdoor test platform to extend validation to true free-space scenarios and confirm the applicability of the proposed linearization method under realistic conditions.

## Figures and Tables

**Table 1:** Comparison of different pre-distortion techniques for FMCW LiDAR.

Ref. Num	[16]	[17]	[26]	[27]	[18]	This Work
Pre-distortion Type	ILC	Iterative Algorithm	Interpolation Linearization	Adaptive Current Injection	Fourier Domain ILC	ILC
Laser Type	DFB	DFB	DFB	–	DFB	DFB
Bandwidth (GHz)	36	26	6.35	23.5	14.07	3.28
Amplitude (V)	0.07	NA	NA	NA	1.8	2
Chirp Duration (μs)	125	500	50	60	5	9.1
Repetition Rate (kHz)	4	1	10	8.3	100	55
Theoretical Resolution (cm)	0.42	0.58	2.36	0.64	1.07	4.57
Measured Distance (fiber)	NA	8 m	100 m	NA	120 m	210 m
$1-r^{2}$	$6\times 10^{-8}$	$5.19\times 10^{-8}$	$9\times 10^{-8}$	$4.1\times 10^{-9}$	$2.21\times 10^{-7}$	$8.1\times 10^{-8}$
Residual Nonlinearity (%)	0.0039	0.005	0.0236	0.0020	0.0128	0.0029

## Data Availability

Data are contained within the article.
